# Mass Casualties and Health Care Following the Release of Toxic Chemicals or Radioactive Material—Contribution of Modern Biotechnology

**DOI:** 10.3390/ijerph8124521

**Published:** 2011-12-07

**Authors:** Ann Göransson Nyberg, Daniela Stricklin, Åke Sellström

**Affiliations:** 1 Swedish Defence Research Agency, FOI CBRN—Defence and Security, 20 Cementvägen, SE 901 82 Umea, Sweden; 2 Applied Research Associates, Inc., 1235 South Clark Street Ste, Arlington, VA 22203, USA; Email: dstricklin@ara.com; 3 European CBRNE Center, KBC Building, 6 Linnaeus väg, SE 901 87 Umea, Sweden; Email: ake.sellstrom@cbrne.umu.se

**Keywords:** mass casualties, triage, biomarkers, biotechnology, diagnostic, chemical, radioactive

## Abstract

Catastrophic chemical or radiological events can cause thousands of casualties. Such disasters require triage procedures to identify the development of health consequences requiring medical intervention. Our objective is to analyze recent advancements in biotechnology for triage in mass emergency situations. In addition to identifying persons “at risk” of developing health problems, these technologies can aid in securing the unaffected or “worried well”. We also highlight the need for public/private partnerships to engage in some of the underpinning sciences, such as patho-physiological mechanisms of chemical and radiological hazards, and for the necessary investment in the development of rapid assessment tools through identification of biochemical, molecular, and genetic biomarkers to predict health effects. For chemical agents, biomarkers of neurotoxicity, lung damage, and clinical and epidemiological databases are needed to assess acute and chronic effects of exposures. For radiological exposures, development of rapid, sensitive biomarkers using advanced biotechnologies are needed to sort exposed persons at risk of life-threatening effects from persons with long-term risk or no risk. The final implementation of rapid and portable diagnostics tools suitable for emergency care providers to guide triage and medical countermeasures use will need public support, since commercial incentives are lacking.

## 1. Introduction—Mass Casualties Following Chemical or Radiological Exposures and the Use of Diagnostic Tools Based on Biotechnology

**Figure 1:**
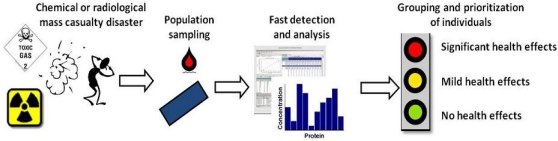
Conceptual flow chart for exposure assessment of mass casualties.

A catastrophic event, involving chemical and/or radiological compounds or devices, may cause mass casualties in the order of several thousand. In the case of individual exposures, any number of resources can be utilized to diagnose and treat a patient (Figure 1). However, in a mass casualty event, the number of potentially affected persons involved will quickly overwhelm the available resources. Therefore, any such disaster would require some form of early screening to accurately and effectively identify and differentiate among patients so that as resources become scarcer they may be used more effectively. It is most critical that those patients likely to develop health consequences, requiring medical evaluation and intervention can be identified. A primary function of such screening could be to sort the unaffected, or “worried-well”, from those patients who soon will truly become symptomatic. Of those likely to become symptomatic, early assessments may also aid in predicting the severity of later health impairments. It may also guide health care personnel (physicians, nurses, *etc*.) to early and effective medical countermeasures and treatments. In addition, initial screening may also predict the likelihood of later, “stochastic” health impairments up to several years after the initial event. Identifying unaffected individuals is also extremely valuable in order to optimize the use of precious medical resources but also because unnecessary treatments can in some cases are deleterious. In accidents involving only a few persons that are treated in local hospitals, follow-up is fairly straightforward. In the mass casualty setting, the massive number of patients will require many local medical resources. Patients may be examined and treated in the field or at any number of different medical facilities in the vicinity, resulting in a significant logistical challenge for patient follow-up and tracking. Triage and early diagnostic screening, however, provides a mechanism for patient tracking for subsequent follow-up.

This review evolves in part from the project Mass casualties and Health care following the release of toxic chemicals or radioactive material, (MASH) supported by Directorate General for Health issues of the European Union, DG Sanco. It summarizes and comments on methods and tools made available from recent advancements in biotechnology to triage mass emergency situations following exposure to toxic chemicals and/or to dangerous radioactive material. Current methods for early diagnostics and exposure assessment are summarized giving particular attention to those methods able to indicate probable health consequences, latent effects, and/or the need for medical treatments. A horizon scan into the fields of molecular diagnostics, proteomics and genomics will are also be made, highlighting particularly promising developments that should be of critical value to the mass emergency situation. The use of electronic, standardized medical algorithms to support medical personnel in the triage situation is reviewed.

### 1.1. C and R Threats and Events

Threat scenarios involving chemicals include the deliberate release of illegally obtained or manufactured chemical warfare agents, the release of purchased or stolen industrial chemicals, and attacks on chemical manufacturing plants, storage sites, or transport vehicles. Moreover, the potential also exists for the malicious use of chemicals to contaminate food or water sources. In the same manner as described for toxic chemicals, radioactive materials may also be used for malicious purposes.

Several industrial accidents causing many casualties highlight the potential impact of a terrorist attack on chemical storage sites or transport vehicles. In 1984, a methyl isocyanate leak at a Union Carbide plant in Bhopal, India, killed as many as 5,000 people and injured more than 14,000 [1]. In the United States since 2002, three major chlorine gas leaks have occurred; one due to a ruptured hose, another due to the rupture of a tanker in a train accident, and the third due to an industrial fire causing several deaths [2]. Explosions in a chemical plant could also disseminate toxic materials into the atmosphere and surrounding grounds, thus causing an environmental health emergency.

The number and variety of different chemicals that pose a health risk to civilian populations is daunting. Terrorists could use any of the traditional chemical warfare agents, ranging from nerve gas and cyanide to pulmonary and vesicating (blister-causing) agents. Whether we like it or not, we must accept the existence of and the risk of chemical warfare agents (CWA) use against the society. The world received a shocking reminder of the potential impact of terrorist use of chemical weapons when the Aum-Shinrikyo sect synthesized the organophosphate sarin and deployed it against civilian targets in Japan 1994 and 1995 [3]. The most serious incident of chemical terrorism until today was the attack in the Tokyo subway in 1995 which resulted in 13 fatalities and approximately 5,000 casualties. For practical purposes the MASH project developed a number of well defined scenarios [4]. The scenarios involve CWA, toxic industrial chemicals (TICs) and radioactive materials and became the standard reference cases against which diagnostic needs were discussed.

Radiological accidents that exemplify the potential impact of a local dispersion of radioactive materials also exist. The 1987 Goiânia accident in Brazil took the lives of four persons with acute radiation sickness and contaminated several hundred persons. More than 100 persons of the 112,000 screened were also internally contaminated [5]. In the Goiânia accident, the radiological agent was cesium-137 in the form of cesium chloride, a radiological material in the form of a salt that is easily aerosolized and dispersed which can result in widespread contamination. Other accidents with industrial radioactive sources have involved cobalt-60 and iridium-192 in the form of metal, although both of them are not that easily dispersed as cesium chloride.

However, a recent radiological accident in Western Delhi (India) involved unshielded cobalt-60 source [6] and is a good illustration of the fact that considerable resources must be spent to recover and safely dispose these types of highly radioactive materials. The MASH project also developed a number of well defined scenarios [4] involving radioactive materials which became the standard reference cases against which diagnostic needs were discussed.

### 1.2. General Principles for Modern Biotechnology Diagnostics

The term biotechnology describes practical applications of the life sciences, ranging from medicine and agriculture to bio-inspired materials. The biotechnology industry grew very rapidly during the 1990s. Part of the reason for this rapid growth was the integration of the life sciences with other enabling technologies such as computers and analytical chemistry by the pharmaceutical manufacturing industry. Due to the announcement in 1999 that the human genome had been sequenced, a new era of biotechnology known as “genomics” was ushered into research and development. In this report we discuss some prerequisites for the implementation of modern biotechnology diagnostics:

- Rapid diagnostic tests must be reliable and easily used in mass casualty situations.
- Immediate as well as long-term effects of exposure to chemicals and radiation must be understood.

### 1.3. Recent Developments in Biotechnology Diagnostics

#### 1.3.1. Genomics

Genomics is a powerful tool to study human genetic variation, which could help identify individual resistance and susceptibility to diseases and responsiveness to medical treatment options. The field includes intensive efforts to determine the entire DNA sequence of organisms and fine-scale genetic mapping efforts. In contrast, the investigation of the roles and functions of single genes is a primary focused of molecular biology or genetics and is a common topic of modern medical and biological research [7].

#### 1.3.2. Transcriptomics

Transcriptomics is the branch of molecular biology that deals with the study of messenger RNA molecules produced in an individual or in a population of a particular cell type. Various methods for reducing the likelihood of false positives or negatives, and new computational approaches are continually being developed. Computer software is commercially available that can perform many of the calculations and data manipulations needed for microarray data analysis [8].

#### 1.3.3. Proteomics

Proteomics is the large-scale study of peptides and proteins, particularly their structures and functions. During recent years, modern high throughput mass spectrometry (MS) together with powerful computational bioinformatics and biostatistics efforts has significantly expanded the capability and impact of proteomics. Many biomedical industrial analysts predict that proteomics will yield additional practical applications, such as drug targets and biomarkers. Ongoing research and development is aimed at further increasing the throughput of proteomics methods [9].

#### 1.3.4. Metabolomics/Metabonomics

Metabolomics is the “systematic study of the unique chemical fingerprints that specific cellular processes leave behind”—specifically, the study of their small-molecule metabolite profiles. Since metabolites are the products and by-products of many biosynthetic and catabolic pathways, metabolomics is now applied to disease diagnosis. Furthermore, it also includes the identification of drugs or chemical exposures [7,10].

#### 1.3.5. Bioinformatics and Systems Biology

Bioinformatics is the application of information technology and computer science to the field of molecular biology [11]. Common activities in bioinformatics include mapping and analyzing DNA and protein sequences, aligning different DNA and protein sequences to compare them and creating and viewing 3-D models of protein structures. The primary goal of bioinformatics is to increase our understanding of biological processes.

#### 1.3.6. Biologically-Based Sensors (Biosensors)

A biosensor is a device for the detection of an analyte that combines a biological component with a physicochemical detector component. The fields of application for biosensors include high throughput screening of pharmaceuticals and the possibility of portable sensors for chemical or other hazards or pathogens [12]. In this paper we discuss the possible use of biotechnology methods for diagnostics after an accident with chemical or radiological agents.

## 2. Human Biomonitoring

Biomonitoring is a valuable tool for assessing human exposures to chemical contaminants in the environment. Biomonitoring tests can be divided into biomarkers of exposure, effect, and susceptibility. In studies of community exposure to an environmental contaminant, biomarkers of exposure are most often used. The ideal biomarker should be sensitive, specific, biologically relevant, practical, inexpensive, and readily available. Seldom does a biomarker meet all of these criteria, and most biomarkers represent a compromise. In designing a community exposure study, consideration should also be given to the selection of the test population, the practicality of collecting biological samples, temporal or seasonal variations in exposure, the availability of background comparison ranges, and interpretation of the test results. Biomonitoring tests provide unequivocal evidence of exposure, but they do not typically identify the source of exposure. Furthermore, rarely do the test results predict a health outcome. For many chemicals, testing must be conducted soon after exposure has occurred. In spite of these limitations, the use of biomonitoring is finding wider application in many scientific disciplines. Recent advances in analytical techniques are expanding the utility of biomarker testing in public health investigations [13].

## 3. Biomarkers

Biological indicators or biomarkers generally include biochemical, molecular, genetic, immunologic, or physiological signals of events in biological systems. The events are depicted as a flow chart between an external exposure to a chemical and resulting clinical effects [14]. 

**Figure 2:**
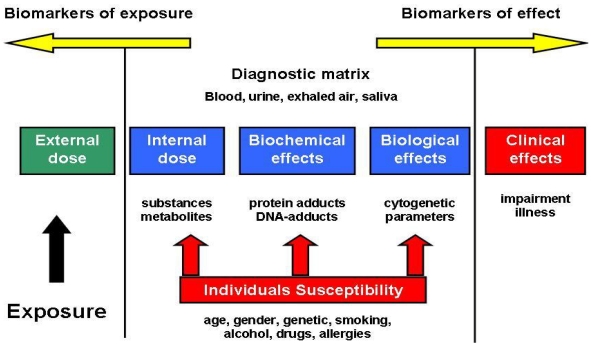
Simplified flow chart of classes of biomarkers [17].

The endpoint of biological monitoring is often referred to as a biomarker, defined as “a change induced by a contaminant in the biochemical or cellular components of a process, structure or function that can be measured in a biological system” [15]. Since biomarkers lie on a flow chart (Figure 2), it may be difficult to delineate between biomarkers of exposure and effect [16].

### 3.1. Biomarkers of Exposure

Biomarkers of exposure have been defined as “a chemical or its metabolite, or the product of an interaction between a chemical and some target molecule or cell that is measured in an organism” [18]. It has been suggested, that an ideal biomarker of exposure should be “specific for a chemical, detectable in small quantities, measured by non-invasive techniques, inexpensive, associated with prior exposure and have an excellent positive predictive value to a specific disease status” [19]. Indeed, it is a hard task to discover biomarkers of exposure for various toxic chemicals that address all criteria above. However, many biomarkers have been identified and used in occupational surveys of exposure to various chemicals [20].

It is generally assumed, that the longer the half-life of a marker, the better it can be correlated with effects resulting from chronic, long-term, low-level exposure to cumulative toxicants. When investigating possible exposure of toxic compounds, the choice of biomarker should depend on how long after suspected exposure the sampling was performed.

#### 3.1.1. Concentration of Parent Chemical in Blood *vs.* Urinary Metabolites

Relevance and stability are perhaps the most important properties making a biomarker suitable for field applications. In this respect, the concentration of the parent compound in biological media is generally preferable to that of metabolites, which can be shared with other substances. Metabolic specificity means that the metabolite is derived exclusively from the parent compound of interest. For some metabolites, the specificity is low since large amounts of these metabolites may be derived from other sources [21].

Therefore, provided that sampling strategies and storage procedures are carefully planned, the parent compound is usually better correlated with exposure as compared to its metabolites [22]. However, the parent compound usually has a shorter half-life, can be volatile, and is usually unrelated to adverse effects, which often occur as a consequence of its biotransformation and metabolic activation. If the focus is on dose-response relationships, urinary metabolites have successfully been used as suitable biomarkers of dose [23].

#### 3.1.2. Adducts to Macromolecules or DNA

The calculated grade of binding to proteins (such as hemoglobin or albumin) or DNA (usually in lymphocytes) is useful as a biomarker, particularly when assessing exposure to genotoxic compounds, because it reflects the dose that has avoided detoxification and reached its target protein or DNA [24]. Because the life span of red blood cells is comparatively long (approximately 4 months), binding to hemoglobin is considered a good biomarker to measure repeated exposure or exposure that occurred weeks or even months before sampling [25].

Albumin has a shorter life-time in blood (20–25 days) and these adducts will thus reflect more recent exposures. One advantage of albumin is that toxic compounds can directly react with this protein when reaching the blood without having to penetrate a cell membrane before forming an adduct [19].

Although macromolecule adducts seem like ideal biomarkers of effect, it should be mentioned that adduct measurements are difficult to perform and to standardize, and are limited to compounds or metabolites forming adducts.

### 3.2. Biomarkers of Effect

A biomarkers of effect has been defined as “A measurable biochemical, physiologic, behavioral, or other alternation in an organism that, depending on the magnitude, can be recognized as associated with an established or possible health impairment or disease” [20]. It has been suggested, that ideal biomarkers of exposure should meet criteria such as non-invasiveness, sensitivity and reflect early responses that precedes functional damage. Analytical methods must be reproducible, easy to perform and applicable to a large number of samples.

Biomarkers of effect may be of value in hazard identification and dose-response assessment. In hazard identification, biomarkers may facilitate screening and/or identification of a toxic agent and characterization of the associated toxicity.

The lack of validation of most biomarkers of intermediate effect is probably the most common argument against broad use of biomarkers in risk assessment. However, efforts to validate biomarkers are rapidly generating a large amount of data measuring intermediate effects occurring after exposure and before illness. Therefore, they should identify early and reversible biochemical events that may also be predictive of later response [23,26]. However, altered physiology is also considered as relevant biomarkers of exposure. The further to the right in the flow chart (Figure 2) the biomarker is, the greater the clinical or health relevance of its measurement. An abnormal value of a biomarker of effect near the centre of the flow chart may not signal negative effects on the health of an individual or group if, for example, the cause is reversible and steps are taken to ensure that the exposure that caused it ceases [15].

Unfortunately, the mechanism of action of many toxic chemicals is unknown at present. Furthermore, there are individual variations in the response to equivalent doses of chemicals. While the outcome of a chemical insult in an individual may be predicted more accurately from biomarkers of effect(s), such biomarkers may not be specific for a single causative agent. Nevertheless, many biomarkers of effect are used in everyday practice to assist in clinical diagnosis. Therefore, most biomarkers of effect have been identified starting from clinical conditions, extrapolating backward to document changes or exposure preceding illness (e.g., early markers of nephrotoxicity that was discovered in the clinic). However, clinical alterations can hardly be interpreted in terms of toxicity in the absence of experimental models, providing some mechanistic clues. Therefore, numerous experimental animal and cellular studies have contributed to the attempts to identify new biomarkers of effect.

Prospective epidemiological studies are also often used for validation of the effect of biomarkers. This type of study provides estimates of the risk of disease of individuals using monitoring of particular biomarkers. Such a strategy is suitable when the outcome is relatively frequent, and the measured biomarker is inexpensive and readily available, e.g., serum cholesterol in cardiovascular disease [27].

### 3.3. Biomarkers of Susceptibility

An individual’s susceptibility to environmentally mediated disease may arise from genetic causes or from non-genetic factors such as age, concomitant disease state, diet, or dietary supplementation.

Genetic polymorphisms may be markers of susceptibility. The rapid advances of the *Human Genome and Environmental Genome* projects are generating a long list of genes and their variants (polymorphisms). Research is helping us to understand which genes are perturbed on the pathway to disease. Many of these genes are quite general in their function and broadly applicable to the assessment of susceptibility. Such genes or groups of genes will, for example, influence or control cell differentiation, apoptosis, cell cycle kinetics, or DNA repair. Receptor-mediated pathways involving alterations in signal transduction can influence a variety of health outcomes. There is a spectrum of human genetic variability such that a distribution of responses to a given exposure can sometimes be predicted.

New technologies such as microchip arrays allow researchers to explore patterns of gene expression. In the face of this burgeoning information and data being gathered by the National Institutes of Health and in other databases, the challenge for researchers interested in environmentally mediated disease or risk assessment is to understand the functionality of genetic polymorphisms and to relate this to disease. We may gain this understanding in humans, particularly by relating laboratory, clinical, and epidemiologic findings. To date, most genetic susceptibility studies have looked at cancers as an end point, although research on other diseases such as asthma is beginning to grow. As our understanding of functionality grows, so will our need for understanding of the ethical implications of our knowledge to individuals and society [15,28,29].

Human Biological Monitoring (HBM) has long been used in occupational health as part of a preventive strategy in the medical surveillance of workers. Currently it is increasingly used as a tool in environmental health policy. More than the classical environmental measurements, pollution gets personal [30]. HBM not only provides valuable information on exposure and its possible effects on health, but also has great impact in raising awareness for possibilities of prevention, and serves as a basis for establishing and evaluating policy measures.

Besides its strong points, HBM clearly has its limitations, including challenging logistical problems and important ethical concerns. Test results are often poorly related to possible health outcomes. They provide only a snapshot of substances present in the body at a single point in time and do not provide information about the source or history of exposure. A long delay may also exist between the availability of test results, the identification of the impact on health and the prospect for measures [30]. Therefore, much work needs to be done, in particular with respect to the proper interpretation of HBM data and its translation into policy actions. Research should allow for a more appropriate use of HBM in the prevention of diseases by further validation of HBM procedures, establishment of relations between biological monitoring parameters and early indicators of disease, development of scenarios for translation into policy, progress in effect monitoring, and provision of better conditions for HBM programs (development of less invasive biomarkers, development of strategies for communication by professionals, setting up the appropriate legal conditions, *etc*.) [31].

The future success depends on a twofold approach: thorough evaluation of the biomarkers that have been found to be useful in various settings [4] and search for new biomarkers [1]. The former is not always easy and hence much attention has been spent on the latter bringing new technologies to bear to look for new biomarkers (see Table 1 and Table 2). A marker may be good in one clinical setting, but not in another. The very best biomarkers will be useful in many contexts. Networks of scientists and clinicians should be used to evaluate multiple biomarkers simultaneously. Another very important feature of the utility of biomarkers is that they must be easy to analyze rapidly and preferably at the bedside if clinical decision-making is to be optimally affected by them. 

**Table 1:** Example of chemicals, biomarkers of exposure and health effects.

Chemical	Biomarker	Sample	Health effects
***Nerve Agents***	Red blood cell or serum cholinesterase EEG changes	Whole blood	Diffuse muscle cramping, runny nose, difficulty breathing, eye pain, dimming of vision, sweating, muscle tremors, loss of consciousness, seizures, flaccid paralysis.
***Cyanides***	Cyanide or thiocyanate	Blood or urine	Giddiness, palpations, dizziness, nausea, vomiting, headache, eye irritation, increase in rate and depth of breathing (hyperventilation), drowsiness, loss of consciousness, convulsions and death.
***Vesicants/Blister Agents***	Thiodiglycol	Urine	Burning, itching, red skin, mucosal irritation, shortness of breath, nausea and vomiting
***Ricin***	Ricinine	Urine, respiratory secretions, serum, and direct tissue	Nausea, diarrhea, vomiting, fever, abdominal pain, chest tightness, coughing, weakness, nausea, fever
***Benzene***	Benzene, Phenol	Blood, exhaled air, urine	Confusion, sleepiness, rapid pulse, loss of consciousness, anemia, damage to the nervous system, suppression of the immune system, carcinogenic, death
***Carbon monoxide (CO)***	Hematologic biomarkers of coagulation and inflammation.	Blood	Tissue hypoxia, headache, nausea, vomiting, dizziness, blurred vision, cardiac arrhythmias, myocardial ischemia, cardiac arrest, hypotension, respiratory arrest, noncardiogenic pulmonary edema, seizures, and coma.
***Nitrogen dioxide***	Nitrate, pentane	Urine, breath	Wheezing, coughing, colds, flu and bronchitis
***Sulfur dioxide***	S-sulfonate	Blood	Lung function changes, life-threatening
***Polycyclic aromatic hydrocarbons (PAHs)***	PAH metabolites	Urine	Pulmonary, gastrointestinal, renal, and dermatologic systems, carcinogenic
***Organic Gases (Volatile Organic Compounds—VOCs)***	VOC’s or metabolites	Breath, blood, urine	Allergic sensitization or asthmatic symptoms, carcinogenic

**Table 2:** Examples of radiation biomarker assays and exposure assessment methods.

*Assay*	Dose range (Gy)	Specificity	Induction time	Persistence	Speed of analysis	Field amenable	Automation potential
***Dicentric Assay***	0.2–5	High	Hours	Several months	2+ days	No	Partial
***FISH***	0.5–5	Moderate	Hours	Years	3+ days	No	Partial
***MN Assay***	0.3–5	Mod.–low	Hours	Several months	3+ days,	No	Yes
***PCC Assay***	ND–20+	High–mod.	Hours	Several months	2+ days	No	Partial
***Gene markers***	Variable, ~0.2–5	Low	Variable Hours–days	Variable Hours—days	<1 day	Possible	Possible
***Protein markers***	Variable, ~0.5–20+	Low	Variable Hours–days	Variable Hours—days	<1 day	Possible	Possible
***Blood cell kinetics***	1–10	Low	Immediate	Up to 2 weeks	Days	Yes	Yes
Bioassay *	<0.001	High	NA	Variable	<1 day	No	Yes

* Bioassay refers to analyzing radionuclides from internal contamination in biological materials and estimation of resulting absorbed dose. ND indicates that a value has not been determined and NA is not applicable.

## 4. Chemical Exposure and Effects on Specific Organs

### 4.1. Chemicals Affecting the Respiratory Tract

Many toxic chemicals can damage the respiratory airways, with potentially life-threatening effects. Ammonia, various alkalis (e.g., bleach and sodium hydroxide), hydrochloric and sulfuric acid, vesicants (e.g., sulfur mustard) and other corrosive agents affect the upper airways, the portion of the respiratory tract that begins at the mouth and nose and ends at the larynx (voice box). Inhalation of these chemicals can cause acute inflammation, painful ulcerations, increased secretions, and difficulties in breathing and swallowing. Secondary bacterial infections may further exacerbate the initial injury. Damage to the upper airway can lead to respiratory failure and death. Exposure can also lead to long-term health problems. For example, chronic respiratory problems, such as scarring and narrowing of the trachea, have been observed in Iranians exposed to sulfur mustard during the Iran-Iraq War of the 1980s (vesicating chemicals will be discussed in more detail in the section entitled “Chemicals Affecting the Skin, Eyes, and Mucous Membranes”).

Some industrial chemicals, including ammonia, chlorine, phosgene, and per-fluoro-isobutylene (PFIB) can cause lower respiratory tract injuries, particularly life-threatening pulmonary edema. Pulmonary edema—the leakage of fluid into the lungs—prevents oxygen delivery to the blood, ultimately preventing oxygen from reaching the brain, kidneys, and other organs. Symptoms may be immediate or delayed; chlorine causes immediate airway irritation and pain, whereas phosgene exposure may not be evident for 24 to 48 hours [32]. People who survive a single, acute exposure to respiratory airway toxicants generally show little or no long-term health problems, although some may eventually develop asthma or chronic bronchitis. Individuals at greatest risk are those with pre-existing heart or lung disease.

#### 4.1.1. Diagnostic Tools

Current diagnostic capabilities are limited. Exposure to chlorine, phosgene, or any of the major alkalis is determined based on clinical signs and symptoms. No screening tests are available to identify individuals exposed to low levels of chemicals.

Mechanistic studies may also aid in the development of new diagnostic approaches. Some chemicals generate metabolic byproducts that could be used for diagnosis, but detection of these byproducts may not be possible until many hours after initial exposure. Additional research needs to be directed at developing sensitive and specific tests to identify individuals quickly after they have been exposed to varying levels of chemicals toxic to the respiratory tract.

One way to approach the diagnostic problems is by development of lung-specific biomarkers. When the integrity of the lung epithelium is broken, lung-specific proteins may leak into the blood circulation. The appearance of such proteins in peripheral blood may indicate lung injury. Clara cell protein 16 and lung surfactant D are two proteins that are produced predominantly in the lungs and therefore have been proposed as biomarkers of public health lung diseases.

Clara cell protein 16 (CC16) is a protein which is secreted in large amounts at the surface of airways from where it leaks into the serum, most likely by passive diffusion. It acts as an immunosuppressant and provides protection against oxidative stress and carcinogenesis. The serum concentration of CC16 is a new sensitive marker to detect an increased permeability of the epithelial barrier, which is one of the earliest signs of lung injury. It has been detected in a variety of both clinical and experimental situations such as exposure to smoke, chlorine, lipopolysaccharides and ozone and is indeed a promising biomarker for this area of research. It is important though to consider that serum levels of CC16 are not specific to one agent, disease state or exposure—it is merely an indication of lung injury [33].

Surface protein D (SPD) is a large, multimeric protein produced mainly by cells of the lungs. It plays an important role in innate immunity and in host defense responses. Over-expression of SPD has been associated with chronic inflammatory conditions such as asthma and interstitial pulmonary fibrosis and chronic obstructive pulmonary disease (COPD) [34].

When exposed to chemical agents it is very likely that the lungs are affected. Inhalation of toxic substances can cause serious injuries both in the acute phase but can also cause chronic damages. It is therefore very important to quickly be able to evaluate the severity of the damage in order to treat it correctly. Common markers for inflammation are cytokines in lung tissue, lavage fluid or serum. By a simple blood sample it is possible to run an assay (ELISA or Bioplex) which gives information on which cytokine levels that are elevated and the inflammation can be treated and monitored over time.

The collection of exhaled breath condensate (EBC) is a simple and non-invasive technique to measure mediators of airway inflammation. It is portable which makes it good for field studies [35]. In EBC exhaled and cooled aerosol droplets serve as seeds for condensation resulting in liquid phase accumulation. EBC does not affect the airway in contrast to bronchial biopsy, bronchoalveolar lavage and induced sputum. There are two groups if inflammatory biomarkers in EBC which are of most importance; eicosanoids and cytokines. Eicosanoids, such as 8-isoprotane, are formed by lipid peroxidation of arachidonic acid during oxidative stress. Cytokines are small proteins involved in mediating inflammation and tissue repair. Cytokine concentrations in EBC samples are usually quantified by enzyme-linked immunosorbent assay (ELISA).

One of the current limitations of EBC measurements is the low concentration of many biomarkers which makes it difficult to perform measurements. It is likely that even more sensitive assays will be able as more potent antibodies are developed and new molecular techniques are introduced [36].

Proteomics, which applies high resolution gel electrophoresis or mass spectrometry (MS) to detect multiple proteins in biological samples, may also be useful to analyze the proteins in EBC.

Exhaled nitric oxide (eNO) is a reliable marker of airway inflammation. The measurement is easy to perform and the result is immediately available. Increased levels of eNO have been measured in patients with respiratory diseases such as asthma and COPD. Therefore it is believed that eNO could be used as a noninvasive biomarker of respiratory inflammation [37]. It is particularly attractive because the test requires little effort from the patient, can be measured even in young children and the result of the test can be immediately available.

### 4.2. Chemicals Affecting the Skin, Eyes and Mucous Membranes

Vesicating agents such as sulfur mustard, nitrogen mustard, lewisite, and caustic industrial chemicals can cause severe blistering and burns to the eyes, mucous membranes, skin, and upper airways, as well as chronic eye inflammation and blindness. The eyes are the organs most sensitive to these chemicals. Vesicants may also affect other parts of the body, including the respiratory tract, immune system, and bone marrow. Sulfur mustard can cause tissue damage within minutes of exposure. Physical injury from other vesicating agents may not be evident for several hours and may result in delayed recognition of exposure [38]. In such situations, an exposed individual may put others at risk of secondary contamination.

The immediate symptoms of exposure to one of these chemicals includes: coughing followed by difficulty breathing/shortness of breath and possibly fluid in lungs within 2–6 hours; burning sensation in the throat and eyes accompanied with watery eyes and blurred vision; nausea and vomiting and developing skin lesions.

The exposure may cause delayed effects even if the person has no clinical symptoms. These includes: difficulty with breathing and shortness of breath and coughing up white to pink-tinged fluid; low blood pressure; heart failure and chronic bronchitis.

#### 4.2.1. Diagnostic Tools

At this time, diagnosis of vesicant injury is based on clinical signs and symptoms and the detection of specific agents in the environment. There are no clinical laboratory tests for sulfur mustard in blood or tissue. However, compounds such as thiodiglycol (TDG) are produced in the body after exposure to sulfur mustard and can be detected in blood, urine, and tissue. Analysis of these compounds requires the use of complex technologies such as gas chromatography-mass spectrometry.

### 4.3. Chemicals Affecting the Nervous System

A variety of chemicals are known to affect the nervous system. Some directly target neural signaling pathways. These include the classic nerve agents (e.g., sarin, soman, tabun, and VX) [39]; organophosphate pesticides [40] and some animal toxins (e.g., botulinum toxin). Chemicals can also affect the nervous system indirectly. For example, metabolic poisons (e.g., cyanide) disrupt cellular respiration, which ultimately prevents the brain from getting sufficient oxygen and energy. Some vesicating agents (e.g., sulfur mustard) appear to have neurological effects as well, although the specific mechanism by which they affect the nervous system is poorly understood.

Neurological symptoms depend on the type of chemical, the level of exposure, and the time elapsed following exposure. Exposure to nerve agents, metabolic poisons, or high levels of sulfur mustard can trigger seizures and loss of consciousness. Other acute effects of nerve agent poisoning include muscle paralysis, cardio respiratory depression, and massive secretion from mucous membranes, eye irritation (miosis), and blurry or dim vision. Other acute effects of exposure to high doses of sulfur mustard include behavioral effects and cognitive difficulties. Nerve agents and metabolic poisons also appear to have serious long-term neurological effects, including neuronal degeneration, but these have not been studied extensively.

The physical states of chemicals that affect the nervous system are an important determinant of the requirements for developing effective countermeasures. Although some chemicals that affect the nervous system exist primarily in the form of a vapor (e.g., hydrogen cyanide), others are oily liquids that are very difficult to remove from the environment and extremely toxic even at miniscule levels (e.g., VX). For these persistent agents, it would be ideal to have pretreatments with long-lasting protective effects that can be administered in advance of possible exposure to personnel who must enter contaminated sites.

#### 4.3.1. Diagnostic Tools

Diagnosis following an acute exposure to a nerve agent is generally based on clinical observations of specific symptoms. Environmental sensors may provide valuable information on probable chemical exposure. One of the greatest challenges in diagnosis is determining whether an individual exposed to a nerve agent is experiencing chemically induced seizure activity in the absence of visible convulsions, since the chemicals that trigger seizures may also cause unconsciousness or paralysis. Sustained seizure activity that is uncontrolled can result in permanent brain injury and death. The standard test for seizure activity involves placing electrodes on the scalp to record electrical activity in the brain using electroencephalography (EEG). Such devices are not portable and have limited practical value in evaluating patients in a mass casualty situation.

### 4.4. Example of Future Diagnostic Tools

#### 4.4.1. Saliva as Diagnostic Tools for Chemical Exposure

Evaluation of saliva can yield information on chemical exposure. Interest in saliva as a diagnostic medium has increased dramatically during the last decade, as saliva and other oral fluids have been shown to reflect systemic fluid levels of therapeutic, hormonal, immunological and toxicological molecules. Oral fluids have also been shown to contain biomarkers associated with infectious and neoplastic diseases. Similarly, the analysis of salivary fluids, like blood-based assays, yields useful diagnostic information for the assessment and monitoring of systemic health and disease states, exposure to environmental, occupational, and abusive substances, as well as for the early identification of harmful agents.

Using sophisticated mass spectrometry equipment, researchers have been able to identify breakdown products of a common pesticide in the saliva of rats exposed to known amounts of the pesticide. The researchers are working now to develop a simpler, portable microanalytical sensor system to quickly diagnose pesticide exposure in humans and a modeling method than can estimate the dose. Researchers say the technology could be adapted to test for a variety of contaminants, including chemical warfare agents. Researchers believe saliva monitoring may be able to detect a broad range of chemical contaminants from ongoing occupational exposure, accidents or even acts of war and terrorism [41].

The need for further research in salivary diagnostics, and advocate that oral fluid-based diagnostics have the potential to provide more accurate and less expensive diagnostic procedures than current methodologies available [42,43].

## 5. Radiation Exposure and Health Effects

Nuclear and radiological scenarios can result in several different types of radiation exposures that range from external exposure to penetrating radiation (such as γ- and x-ray sources) to exposure from internalized radionuclides. The primary concern from α-emitting radionuclides is internal contamination since the range of the α-particle is very short and cannot penetrate the dead layer of the skin but can deliver a significant dose to tissues in the body if internalized. The range of β-particles is longer and dependent on the energy of the emission. If deposited on the skin, radiations burns can result from the energy deposited and absorbed in the skin and β-emitting radionuclides pose a significant health hazard if internalized. The impact of γ- or x-ray emitting nuclides, or penetrating radiation, depend on the activity of the source. High activity sources can result in cutaneous effects, which are often localized. Some dose can be received from internalized γ-emmitting nuclides, however, the primary concern in most scenarios is from high doses received externally.

### 5.1. Radiation Injury

A whole body, or significant partial body, external exposure to penetrating radiation (>1 Gy) delivered in a short time from, *i.e.*, at a high dose rate, will result in specific signs and symptoms termed acute radiation syndrome (ARS) [44]. The four phases of ARS include prodromal, latent, manifest, and recovery or death. The prodromal phase occurs in the first 48 hours and occasionally up to 6 days. The latent phase is characterized by a short, transient period of time (few days to a few weeks) where patients demonstrate some recovery by being less affected by symptoms. Manifest illness then follows which may continue for several weeks, and is dominated by immunosuppression. The final phase will be recovery or death, dependent on the dose and other factors such as the individual’s age, existence of other injuries, *etc*.

The focus of this discussion is primarily on acute, high dose exposures from external sources and the acute effects observed after such exposures. However, it is important to note that other injuries and latent effects can occur from radiation exposure. In the case of internalized radionuclides, specific organ damage can occur to any organ in which the radionuclide accumulates in or is retained in. For example, α-emitting, insoluble plutonium and americium radionuclides can be retained in the lungs for a long time after inhalation exposure. Once incorporated into the body, they accumulate in the bone. Therefore, these nuclides can significantly impact pulmonary tissue, resulting pneumonitis or fibrosis, and the red bone marrow resulting in impaired hematopoietic function. Latent effects that can result from radiation exposures include cataract formation and increased cancer risk.

### 5.2. Organ System Effects

Specific symptoms, their onset and severity are highly dependent on absorbed radiation dose. Proliferating cells are most sensitive to radiation and hence exhibit acute effects. As such, hematopoietic stem cells and intestinal crypt cells are inherently sensitive and result in clinical effects that predominate in a predictable range of doses [45]. The specific syndromes manifested in ARS include the neuro/cerebrovascular, hematopoietic, gastrointestinal, and cutaneous systems.

The neurovascular or cerebrovascular system will exhibit symptoms, the degree of which varies depending on the dose received. In fact, the onset of nausea and vomiting has been used to estimate dose and is well correlated. Fatigue, fever, headache, hypotension, as well as neurological and cognitive deficits can be observed [46]. At very high doses (≥30 Gy), cerebrovascular syndrome occurs. Immediate nausea, vomiting, hypotension, ataxia, and convulsions may occur soon after exposure, followed by unconsciousness and death within days [47]. The origin of some of these symptoms relate to changes in permeability in the blood brain barrier and vasculature system.

Symptoms observed in from the gastrointestinal system include diarrhea, abdominal cramps, and pain resulting from losses in the mucosal layer in the gut. Gastrointestinal syndrome occurs with doses in the range of 6–20 Gy due to death of the intestinal stem cells causing hemorrhaging. In this dose range, prompt onset of nausea, vomiting, and diarrhea will be observed followed by recurrence of gastrointestinal symptoms after the latent phase. Sepsis can occur from gut flora leaking into the systemic circulation. Intestinal bleeding, electrolyte imbalance, and death may follow, usually in 8–10 days.

The hematopoietic system will exhibit in general an initial rise in some cells, followed by a decline. Lymphocytes, granulocytes, and thrombocytes are all affected. The depletion of neutrophils will result in increased susceptibility to infection while thrombocyte depletion can result in hemorrhaging. Hematopoietic syndrome occurs at whole body doses of 2–10 Gy due to bone marrow depression and is most strikingly characterized by lymphocyte depletion. Blood cell depletion kinetics can be used to indicate dose and facilitate prognosis. Many persons can survive ARS if treated with fluids, antibiotics, and blood products. If standard care is performed, death generally results only when there are no surviving stem cells in the red bone marrow.

The cutaneous system also exhibits effects after radiation exposure [48]. Onset and degree of erythema and epilation has been correlated with radiation dose. At very high doses, swelling, blistering, and desquamation can occur. While cutaneous syndrome is not generally a stand-alone mechanism of mortality, it contributes the overall systemic response to radiation and has been implicated in contributing to multi-organ failure [49]. Furthermore, radiation burns from localized radiation deposition on the skin significantly contributed to the observed mortality in Chernobyl victims.

With successful treatment of high dose radiation exposure and ARS, late effects may be observed in other organs. For example, the lungs and kidneys exhibit damage months after the exposure due to chronic inflammation leading to fibrosis or necrosis. Some cases can result in multi-organ dysfunction and failure.

### 5.3. Diagnosis and Assessment

The appropriate treatment of radiation injury is highly dependent on accurate diagnosis and assessment. Current methods used for determining radiation dose include any physical dose estimates or reconstruction of the exposure, clinical signs and symptoms, blood cell kinetics, and cytogenetic biodosimetry. A simple system for using the collective diagnostic information to rapidly and reliably classify acute radiation injury for managing treatment, termed Radiation Injury Severity Classification (RISC), has been described [50]. The system was adapted from previously described injury groups [51] to address special concerns in management of mass casualty events. A similar approach for medical radiation assessment and management which extensively describes diagnostic approaches based on organ specific parameters has been developed as part of an EU project termed METREPOL (Medical Treatment Protocols for Radiation Accident Victims) [46].

Radiation assessment is only one part of the overall decision for treatment of an individual. Subsequently, all available knowledge concerning the affected persons(s) such as the existence of other injuries, age, health status, and dose will be used in determining eventual treatment and therapy.

#### 5.3.1. Signs and Symptoms

The onset of clinical signs and symptoms can be reliably used to assess the extent of radiation dose (see Table 3). Some of the symptoms expected after significant radiation exposure (>1 Gy) include headache, nausea, vomiting, and diarrhea. At very high whole body or local skin doses (>6 Gy), cutaneous effects such as erythema can be observed, as well as seizures and incapacitation above 10 Gy. Of these symptoms, the most useful and reproducible parameter for assessing dose is the time to emesis or vomiting. A very high dose is assessed with onset of these symptoms within one hour, and significant dose with onset within 2–4 hours. However, the exact time of exposure must be known in order to accurately determine the lapse time before onset. In accident scenarios and in the presence of other significant injuries, *onset of vomiting* may be influenced by shock and trauma. In this case, a reliable dose estimate may not be possible based on this parameter. The following assessments are primarily valid for whole body doses [45,52,53].

**Table 3:** Signs and symptoms correlated with absorbed radiation dose [54].

	Onset of Symptom				
ARS/ Dose	Vomiting	Diarrhea	Headache	Consciousness	Medical care
Mild (1–2 Gy)	>2 hr	-	Slight	-	Outpatient
Mod. (2–4 Gy)	1–2 hr	-	Mild	-	Hospital
Severe (4–6 Gy)	<1 hr	Mild, 3–8 hr	Mod., >24hr	-	Special hosp.
Very sev. (6–8 Gy)	<30 min	Heavy, 1–3 hr	Severe, 3–4 hr	Possible	Specialized hospital
Lethal (>8 Gy)	<10 min	Heavy, <1 hr		Sec.–minutes	Palliative care

#### 5.3.2. Blood Cell Kinetics

Since the hematopoietic system is one of the more sensitive organs to radiation, blood cells parameters can be valuable tools in estimating dose. However, the methods discussed again apply primarily to whole body exposures and assessments will not be valid for partial body exposures [55]. These endpoints are further limited by the natural variability in individual baseline cell counts and sensitivities.

If initiated early, serial blood cell counts can provide a reasonable estimate of dose as well as an indication of the treatments needed and eventual prognosis. A specific pattern of blood cell changes may be observed in the first couple of weeks after exposure and can help to provide a clear picture of the hematological response [56]. These changes involve decreases and increases in granulocytes, platelets, and lymphocytes. Granulocytes may transiently rise before neutropenia (severe decrease of neutrophilic granulocytes) begins [44]. In some cases, granulocytes may experience an abortive rise around day 5–10, which is indicative of residual, viable hematopoietic tissue capable of reproducing granulocytes. Lymphocytes decline rapidly and later repopulate if the radiation dose is not too extreme where bone marrow or stem cell transplantation is warranted. Platelets may have an initial shoulder before decline or begin a progressive decline within the first week depending on dose [57].

The parameter most often used and recommended for aiding dose estimation is the kinetics of lymphocyte depletion. In the best case, complete blood cell counts with determination of the leukocyte differential are obtained immediately and followed up 2–3 times per day over the first week [45]. The rate of the lymphocyte decline is highly dependent upon the radiation dose received and can be used to estimate exposure based on the slope obtained from the differential counts. Ideally, blood cell counts are followed until the nadir in the neutrophil count is established. In assessment of blood cell kinetics, some researchers recommend considering both lymphocyte and neutrophil counts or their ratio as an indicator of dose [50].

Utilization of additional hematological information (*i.e.*, granulocyte, lymphocyte, and platelet counts) affords a better diagnosis and facilitates prognosis since the changes observed indicate the effect on the blood stem cell pool which can aid the prediction of eventual recovery or existence of irreversible injury [57]. If irreversible injury has occurred, definitive care such as stem cell replacement is warranted if other injuries are minimal and the dose is not too great.

#### 5.3.3. Biodosimetry

The most established method used as a radiation biological dosimeter is cytogenetic analysis of chromosomal aberrations by the measure of dicentric aberrations in metaphases from blood lymphocytes (see Table 4). 

**Table 4:** Dicentric chromosomes frequency expected in human lymphocytes resulting from different radiation doses [45].

Dose Estimate	Dicentrics in peripheral blood lymphocytes	
	Per 50 cells (triage)	Per 1000 cells
0	0.05–0.1	1–2
1	4	88
2	12	234
3	22	439
4	35	703
5	51	1024

The dicentric assay is currently the gold standard in biodosimetry [58]. A blood sample should be obtained at least 24 hours after exposure, followed by lymphocyte cell culture, and subsequent analysis. The time from sample acquisition to dose estimate is typically 2–3 days. If the radiation event involves a large number of individuals, analysis on a triage basis may be performed. This method evaluates fewer cells in the analysis to provide a more general estimate of dose category. The dicentric frequency observed in the patient is standardized by an *in vitro* dose-response curve specific for different types of radiation. The frequency of dicentric aberrations is well correlated with dose and may be used to prepare a dose estimate. Over-dispersion of aberration frequency can be used to provide an estimate of partial body dose with the use of mathematical models. However, for certain inhomogeneous exposure events, cytogenetic dosimetry is ineffective. Examples of such situations are exposure to β-emitting radionuclides deposition on the skin and incorporation of many radionuclides that accumulate in specific organs and tissues. Altough internalized caesium-137 has more or less uniform distribution in the body and may be an exeption [6,59].

The dicentric assay is reliable only up to 5 Gy due to cell death and saturation of the dose response curve at higher doses [60]. Another cytogenetic assay using chemical phosphatase inhibitors to induce premature chromosome condensation (PCC) has been applied reliably at doses up to 20 Gy [61], the utility of which was demonstrated during the Tokai-mura criticality accident [62] and provides a good alternative for evaluation of higher doses of radiation exposure. The PCC assay can provide valuable complementary information for medical treatment decisions since with present advances in modern medicine, the critical decisions concerning definitive care in acute radiation injury is in the dose range above 5 Gy.

Other cytogenetic methods include the micronucleus assay and analysis of stable translocations by fluorescence *in situ* hybridization (FISH). Micronuclei are formed when chromosomes fail to segregate properly at mitosis and appear in the cytoplasm. The method requires a longer incubation time than the dicentric assay but requires less analysis time because the cells are easier to evaluate. At low doses, the micronucleus method is uncertain, since micronuclei also are produced by other clastogens resulting in a higher background frequency. Additionally, coalescence may occur at high doses causing a flattening of the dose-response curve [63]. Fluorescence *in situ* hybridization (FISH) has been developed for retrospective analyses to complement the shorter halflife dicentrics in the lymphocyte pool. FISH proved efficacious in detecting stable translocations between chromosomes [64]. Persistent translocations may provide a retrospective estimate of stem cell dose many years later [65]. Its value has been demonstrated when significant time has elapsed between radiation exposure and the discovery of that exposure [54,66,67].

#### 5.3.4. Dose Reconstruction

In many cases, physical dose estimates are not available or will be only a rough estimate for a single individual involved in a large accident. However, when available and when time allows, physical dose reconstruction of dose may be very valuable since eventual prognosis concerning whole body radiation exposure is highly associated with the absorbed dose estimate. In some cases, dose reconstruction methods can be applied to estimate the partial body equivalent dose when partial body exposures have occurred or in cases where part of the body has been shielded. This greatly facilitates medical management decisions since shielding of the lung or essential parts of the bone marrow will greatly impact the chance of survival for an exposed individual.

#### 5.3.5. Future Diagnostics

Unfortunately, the methods described above are inadequate for the diagnostic evaluation of radiation exposure in a mass casualty scenario. None of the methods are suitably fast, high-throughput, or field amenable for use in the field for triage sorting and application for making emergency care decisions. However, several methods currently under development may be able to address this gap.

The γH2AX foci analysis is based on measurement of a histone-related protein that forms a variant form of H2A, which codes the DNA [68]. Double strand breaks in the DNA caused by ionizing radiation results in phosphorylation of H2AX adjacent to the number of double strand breaks [69]. Antibodies specific fluorescent staining of the phosphorylated form of H2AX, termed γH2AX foci, allows observation and quantification of DNA damage. There is a good relation between γH2AX foci and DNA damage up to ~5 Gy. Currently, γH2AX foci are counted manually by microscopic analysis, but efforts are underway to develop high throughput analyses, potentially using flow cytometry and other means.

Gene expression signatures are also a potential alternative for radiation dose assessment. Microarrays embedded on chips for genomic profiling may afford a potentially fast and high-throughput technique. This procedure monitors the gene expression profile of cells that can detect 2–3 fold changes in gene expression between samples. The specific genes which expression changes following radiation exposure have been identified. Recent work suggests that the technique has potential applicability for dosimetry estimations [70,71,72,73,74]. A linear dose-response for induction of several genes has been observed as low as 0.02 Gy. In a recent study on human peripheral blood from healthy donors, a 74-gene signature was identified that distinguishes between radiation doses (0.5–8 Gy) and control. Expression patterns of five genes (CDKN1A, FDXR, SESN1, BBC3 and PHPT1) from this signature were also confirmed by real-time PCR. The authors were able to on a single set of genes, predict radiation dose at both 6 and 24 hours after exposure without the need for pre-exposure sample, which is an important advance for gene expression biodosimetry. The separation by exposure dose was clearest between the lower doses with some overlap evident between the highest doses of 5 and 8 Gy. An effect of time since the radiation was also evident in the 74-gene signature. Inter-individual variation is another important concern for the development of gene expression biomarkers. Within a set of ten donors used by Amundsen *et al*, variations by radiation exposure dose were greater than the variations in expression between donors, allowing relative accurate classification of samples by dose. In a recent study of radiation induced gene expression in human peripheral lymphocytes using real-time PCR, there was a minimal variance of base line expression and consistent radiation responses among five genes between 20 healthy donors [75]. Gene expression signatures are looking increasingly attractive as potential biodosimeters for radiation exposure. However, further validation in terms of *in vivo* responses in cancer patients and animal models, inter-individual variability, radiation specificity of the signatures is still needed. In order to make gene expression signatures practically useful for mass casualty screening, they will also be needed to be exported from the laboratory-based microarray platforms currently used for discovery research to higher throughput forward deployable platforms. Approaches have been suggested based on q-RT-PCR [76] and nano-technology “lab-on-a-chip” designs [77].

Similar to gene expression profiling, proteomic and metabolomic profiling also hold promise as alternative methods for radiation dose assessment. To exploit current advances in the ability to monitor changes in protein expression to identify a biomarker radiation exposure, it is necessary to quantify the protein expression changes and relate them to dose or establish dose-dependent panel of such changes. A number of techniques are available to examine protein profiles including higher resolution surface-enhanced laser desorption ionization time of flight mass spectrometry (SELDI-TOF MS), [78]. The approach has been tested in cancer patients before and during radiotherapy and the exposed population could be distinguished from the unexposed population with high sensitivity and specificity. In the analyses 23 protein fragments/peptides were uniquely detected in the exposed group. This shows that the protein profile in serum changes following radiation exposure in a manner that is probably dose-dependent. The approach requires further development and a defined dose-response relationship remains to be determined. Identification of radiation-induced metabolic changes and an understanding of the signaling pathways involved are necessary for development of a reliable metabolomic marker to assess radiation exposure and extent of injury. Using state-of-the-art HPLC MS (TOF), the entire human metabolome has been illustrated as a feasible compartment to analyse the metabolome with respect to radiation dose.

### 5.4. Triage

An initial assessment is required to establish the necessary treatment protocol for injured patients involving radiation. Triage is performed so that patients may be sorted according to the urgency of care required. In mass casualty situations, patients are further sorted to optimally match patient needs to limited resources.

#### 5.4.1. Radiation Independently

When radiation injury is encountered without other types of wounds, triage is focused on diagnosis and establishment of dose estimate. If any internal radiation contamination exists, decontamination and decorporation treatment should begin as early as possible since some nuclides can become lodged in tissues. The specific therapies for patients after a radiological accident are highly dependent on the dose and overall patient assessment. However, in general, the hematopoietic syndrome and resulting immune-suppression are the immediate priorities in treatment of radiation-only injuries. Treatment of hematopoietic syndrome involves predominately administration of fluids and antibiotics. The use of cytokines to stimulate hematopoietic cell proliferation has been proposed but must be initiated within the first 24–48 hours to be effective. At higher doses gastrointestinal effects can be observed but few treatment options exist for these effects. The final dose cut-off for effective treatment is in the dose range where eventual fibrosis of the lung will evolve (~10 Gy).

#### 5.4.2. Combined with Trauma and/or Wounds

In many scenarios, radiation injury will be accompanied by burns, wounds, and trauma resulting from a blast. During the triage stage, all life threatening injuries should be prioritized and handled first. If a contamination is an issue after a patient is stabilized, decontamination and decorporation therapy can be considered. Thereafter, burns and wounds should be prioritized. If surgery is required, this should take place in the first two days after exposure. Establishment of radiation dose during this period will indicate the need for other measures such as supportive care and in some cases isolation. The prognosis of irradiated patients with combined injuries is much poorer than those with radiation alone. The changes in the traditional triage classification when combined with radiation are shown in Table 5.

**Table 5:** Triage categories for combined injuries dependent on radiation dose [45].

Conventional triage category	Changes in triage category with whole body radiation		
	<1.5 Gy	1.5–4.5 Gy	>4.5 Gy
Delayed	Delayed	Variable	Expectant
Immediate	Immediate	Immediate	Expectant
Minimal	Minimal	Minimal	Minimal
Expectant	Expectant	Expectant	Expectant
Absent	Ambulatory monitoring		

### 5.5. Emergency Care

After an initial triage, patients should be sorted into treatment categories with patients having exposures less than 1 Gy not normally requiring further treatment and those with greater than 10 Gy receiving supportive and palliative care since fatal outcome is anticipated. In general, this stage of care is for patients that have received significant but non-lethal doses of radiation. Treatment actions in this phase generally address the symptoms resulting from hematopoietic syndrome.

#### 5.5.1. Diagnostic Tools

Normally the presence of a contaminating radionuclide (a gamma and/or beta emitter) would be quickly identified, using portable radionuclide detectors handled by radiation safety officers. Individuals with observed external contamination would be quickly decontaminated by removal of clothes and washing. Internal contamination as assessed by portable detectors or whole body counting. Patient samples would be taken for urgent gamma spectrometry analysis at specialized laboratories (responses within hours to days). Selection of individuals for monitoring, including clinical follow-up and biological dosimetry, would be considered primarily for patients showing clinical symptoms indicative of “acute radiation syndrome”. Responses to “classical” biodosimetry with dicentrics would take days to weeks. Dose action levels (upper and lower) regarding internal contamination would be considered, evaluating whether to initiate medical treatment to reduce doses (e.g., through reduced decorporation by decreased absorption and/or increased excretion; drugs like ferric ferrocyanide—“Prussian blue”).

For individuals exposed to a sealed source of ionizing radiation, *i.e.*, no contamination exists.there is a need for extensive biological dosimetry involving accredited and novel methods (see above) combined with repeated clinical assessments, particularly of individuals presenting with the “acute radiation syndrome”. Both cases illustrate the need for novel, rapid, high throughput, accredited biotech solutions, particularly in the mass casualty setting. For guidance references, see TMT Handbook [79], including sub references from e.g., various ICRP publications.

## 6. Conclusions

**Figure 3:**
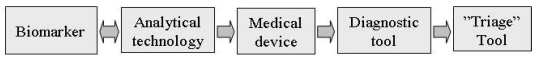
The intrinsic logistics of such development towards different diagnostic tools is simplified in the block diagram below.

Chemical or radiological mass casualties call for rapid and robust diagnostic technologies (Figure 3). These should determine, monitor and assist in handling of the exposed. As indicated in this review the development of new and improved medical technologies utilizing recent developments within medicine, biotechnology and intelligent communication technology (ICT) is foreseen. New insights into useful biomarkers and the development of useful analytical technologies or concepts are reciprocally dependent and driven by “curiosity” or scientific incitements. Sometimes the scientific push giving new biomarkers or new analytical technology also results in medically useful devices.

These devices are basically analytical instruments calibrated and somewhat modified to better serve their intended medical purpose and to be a useful property of the advanced hospital laboratory. Diagnostic tools as indicated in the figure, refers to mass produced (*i.e.*, relatively cheap) simplified instruments that may serve as a standalone capability in acute care or in primary medical care. These are dedicated to simple routine analyses, such as blood glucose assessments. Finally, the triage tool is the instrument similarly dedicated to emergency medicine and mass casualties. It must be robust, simple, efficient and cheap in order to be produced and implemented. The development of diagnostic and/or triage tools will depend on commercial realities. If there is a market, these tools will eventually appear when the technological advancements so allow. If the market is lacking, which is the case for such rare events as mass emergencies, such development has to be politically augmented and realized through public investments.

The present review deals with the prospect of and need for diagnostic or triage tools when handling mass casualty situations following the exposure to toxic chemicals or radiation. The development of diagnostic principles for radiation damage and/or signs of intoxication have been dealt with in parallel. Obviously, the support and need for diagnostic tools within such professions as nuclear medicine, hematology and oncology has been larger than the corresponding support for development of diagnostic tools for intoxication. Biomarkers and diagnostically useful methods have, therefore, been developed for the assessment of dose effects and radiation injuries. None of these methods have as yet become a candidate for triage tool development. The development of diagnostic tools for intoxication is lagging behind. There may have been less support and/or need for this development and, indeed, there are less professionals involved in issues of intoxications in a clinical setting, *i.e.*, involved in triage like situations.

This review underlines the relative maturity of the underpinning science, *i.e.*, about biomarkers, analytical procedures and miniaturization. The challenge now is to develop diagnostic assays, diagnostic tools and triage tools based on bodily fluids and/or cells that assess chemical exposure or already absorbed doses of ionizing radiation or deposited internal radiological contamination and simplified and miniaturized into useful triage tools. At the same time, diagnostic technologies assessing physiological functions that are altered by e.g., chemical or radiological exposures has to be developed as an integral tool in the medical management of casualties. In the end this becomes a question of resources and priorities. Making our society more resilient towards accidental or intentional release of toxic chemicals or radioactive material has to become an issue of high priority.
